# Measurement of 3-acetyl-11-keto-beta-boswellic acid and 11-keto-beta-boswellic acid in *Boswellia serrata* Supplements Administered to Dogs

**DOI:** 10.1186/s12917-019-2021-7

**Published:** 2019-08-01

**Authors:** Erin Miscioscia, Justin Shmalberg, Karen C. Scott

**Affiliations:** Department of Comparative, Diagnostic, and Population Medicine, College of Veterinary Medicine, University of Florida, Gainesville, FL USA

**Keywords:** *Boswellia serrata*, Boswellic acids, Frankincense, Chromatography, Nutraceutical, Canine osteoarthritis

## Abstract

**Background:**

Osteoarthritis is a common canine disease frequently treated with nutritional supplements that often lack independent verification of ingredients, active ingredient concentration, efficacy, or safety. Human supplements containing *Boswellia serrata* extracts (BSE) with high concentrations of active constituents 3-acetyl-11-keto-β-boswellic acid (AKBA) and 11-keto-β-boswellic acid (KBA) are bioavailable, safe, and efficacious in the alleviation of symptoms of naturally occurring osteoarthritis in people. Thus, oral AKBA and/or KBA supplementation could be a promising novel therapy for dogs with osteoarthritis. The primary objective of this study was to determine the concentrations of AKBA and KBA within six human and seven canine market formulations containing BSE administered to dogs, using a derivation of the previously validated high performance liquid chromatography (HPLC) method. The secondary objective was to compare measured concentrations to label claims.

**Results:**

The mean concentrations of AKBA and KBA within the formulations tested were 42.3 mg/g AF (0.1–155.7 mg/g AF) and 5.2 mg/g AF (0–24.8 mg/g AF), respectively, with four of the formulations containing an undetectable amount of KBA. None of the market formulations had a label claim for KBA. For the five tested formulations with a label claim for AKBA, the mean percentage of detected AKBA was 173% of the concentration listed on the label (range: 114–224%). Formulations claiming to contain AKBA had a mean AKBA concentration of 98.2 mg/g AF, significantly higher than formulations claiming only to contain BSE (7.4 mg/g AF; *p* = 0.01).

**Conclusions:**

This study demonstrated a large variation of boswellic acid concentrations in market formulations claiming to contain BSE, with products claiming to contain AKBA containing higher concentrations of AKBA than other products. There was also a large variation in, and overall high, percent difference between label claims and measured concentrations of AKBA. All products met or exceeded label claims. However, differences between label amounts and detected concentrations confirm the need for independent laboratories to quantify concentrations of active ingredients in supplements containing BSE. This would be necessary prior to the use of these formulations in the research or clinical setting.

## Background

The oleogum resin from the bark of the *Boswellia serrata* tree, popularly known as Indian Frankincense, has been used for centuries in Ayurvedic medicine as an anti-inflammatory agent [1, 2]. It has been determined that the anti-inflammatory activity is primarily due to two pentacyclic triterpenic acids [3-acetyl-11-keto-β-boswellic acid (AKBA) and 11-keto-β-boswellic acid (KBA)] [1, 3]. These active boswellic acids act primarily to inhibit 5-lipoxygenase and subsequent leukotriene production, but also reportedly inhibit nuclear factor-kappa B activation and tumor necrosis factor alpha generation, thus providing a multimodal anti-inflammatory effect [1, 3]. BSE is, however, complex in composition, with a multitude of components, such as beta-boswellic acid and flavonoids, that could be providing complementary anti-inflammatory effects that have not yet been fully elucidated [4–6].

Many human and canine market formulations containing *B. serrata* extracts (BSE) currently exist, the majority of which purport to benefit humans or dogs suffering from osteoarthritis. However, due to a lack of pre-market clearance requirements in the United States, the majority of these formulations lack proof of ingredients, active ingredient dosage, proof of efficacy, and/or proof of safety [7]. Pharmacokinetic profiles and safety of oral administration of BSE have been established in humans, though research remains ongoing to determine the most bioavailable form of BSE to enhance pharmacokinetic profiles [8–13]. Additional studies have demonstrated efficacy of oral AKBA administration in the alleviation of symptoms of naturally occurring osteoarthritis in people [10–12]. One non-placebo controlled study demonstrated a reduction in clinical signs of naturally occurring osteoarthritis in dogs supplemented with an oral BSE formulation [14]. Thus, oral BSE formulations could be a promising novel therapy for osteoarthritis in dogs. However, efficacy, safety, and dosing of BSE formulations, specifically the dosing and pharmacokinetics of the most active boswellic acids, must be established prior to widespread use for the treatment of canine osteoarthritis.

One study [15] previously validated a high performance liquid chromatography (HPLC) method for the determination of AKBA and KBA concentrations in market formulations and a second study [16] used liquid chromatography and mass spectrometry to determine total boswellic acid content of seventeen market formulations and AKBA content of five market formulations administered to people. The primary objective of the current study was to determine the concentrations of AKBA and KBA within six human and seven canine market formulations containing *B. serrata* extract which are administered to dogs, using a derivation of the previously validated HPLC method [15]. The secondary objective was to compare measured concentrations to label claims. It was hypothesized that for formulations with AKBA concentration claims on the labels, the estimated AKBA concentrations detected would yield different concentrations than those claimed on the labels, and also hypothesized that the mean concentration of AKBA in products claiming AKBA would be higher than products claiming only BSE.

## Results

### Validation of the derived method

Retention times were 5.2 min for AKBA and 3.1 min for KBA, similar to those described in a previous study [15]. The correlation coefficients (R^2^) for the calibration curves of AKBA and KBA were 0.9955 and 0.9907, respectively. Inter-assay precision (% difference from the mean) ranged from 0.26–3.67% and 0.12–2.77% for AKBA and KBA, respectively. Intra-assay precision (% difference from the mean) ranged from 0.17–3.82% and 0.11–2.87% for AKBA and KBA, respectively. Recoveries from spiked herbal samples were obtained with a mean of 102.8±5.7% for AKBA and 100.3±3.1% for KBA. The limit of detection for AKBA was 0.12 μg/mL and for KBA was 0.11 μg/mL.

### Analysis of the market formulation

**Fig. 1:**
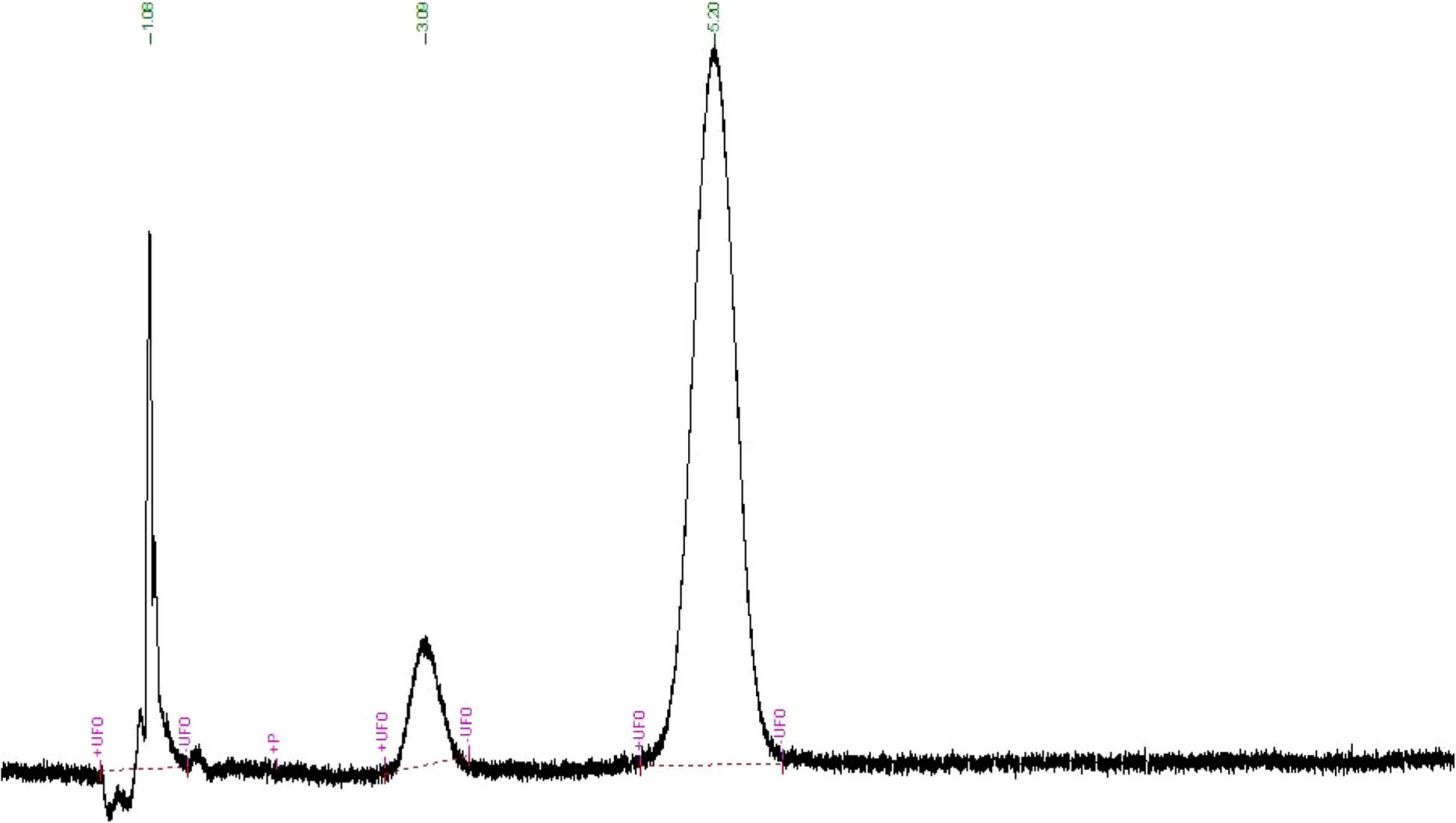
Example chromatogram from Human Market Formulation 4. Peak associated with KBA at 3.1 min and peak associated with AKBA at 5.2 min

The derived HPLC method was used to estimate AKBA and KBA concentrations within six human and seven canine market formulations (HMF and CMF) claiming to contain BSE (Table 1). No interfering peaks were detected in chromatograms, indicating that other compounds within the formulations did not interfere with the estimation of boswellic acids (Fig. 1: Example Chromatogram from HMF 4). The mean concentrations of AKBA and KBA within the market formulations tested were 42.3 mg/g AF (0.1–155.7 mg/g AF) and 5.2 mg/g AF (0–24.8 mg/g AF), respectively, with four of the formulations containing an undetectable amount of KBA.

**Table 1:** Concentrations of 11-keto-β-boswellic acid (KBA) and 3-acetyl-11-keto-β-boswellic acid (AKBA) in Canine and Human Market Formulations Administered to Dogs

Product	[Mean KBA] Detected (mg/g)^§^	[Mean AKBA] Detected (mg/g)^§^	[AKBA] Label Claim (mg/g)^§^	Detected AKBA as % of Label Claim
CMF 1^‡^	2.0	2.5		
CMF 2^‡^	24.8	8.8		
CMF 3^‡^	8.9	0.1		
CMF 4^‡^	0.3	0.4		
CMF 5^‡^	0.02	1.7		
CMF 6^‡^	0	2.7		
CMF 7^†‡^	0.6	56.2	49.4	114
HMF 1^†‡^	0.1	12.7	7.1	180
HMF 2^†‡^	0	118.4	64.6	183
HMF 3^†‡^	0	147.9	66	224
HMF 4^‡^	15.7	23.5		
HMF 5^‡^	14.8	19.2		
HMF 6^†‡^	0	155.7	94	166

† Formulations with a label claim for concentration of AKBA

‡ Product names and manufacturers for the market formulations tested: CMF 1: Advanced Hip & Joint, Springtime Inc., Cockeysville, MD; CMF 2: *Boswellia* Herbal Joint Support, Platinum Performance, Buellton, CA; CMF 3: S.O.D. & *Boswellia* Extra Joint Support, NaturVet, Temecula, CA; CMF 4: Synovi G4 Soft Chews for Dogs, Bayer, Shawnee Mission, KS; CMF 5: Cosequin Std Strength Plus *Boswellia* & HA Chewable Tablets, Nutramax Laboratories Veterinary Sciences, Inc., Lancaster, SC; CMF 6: Dasuquin Advanced Small to Medium Dogs Chewable Tablets, Nutramax Laboratories Veterinary Sciences, Inc., Lancaster, SC; CMF 7: Maximus Joint Booster (UC-II), PETdiatric Laboratories, Selangor, Malaysia; HMF 1: Flex-a-min Triple Strength with Joint Flex, Nature’s Bounty, Bohemia, NY; HMF 2: Synergy 5-Loxin *Boswellia serrata* Extract, Vitacost, Boca Raton, FL; HMF 3: 5-LOX Inhibitor with Apresflex, Life Extension, Fort Lauderdale, FL; HMF 4: *Boswellia* Extract 500 mg Capsules, NOW Foods, Bloomingdale, IL; HMF 5: Organic Frankincense Powder, Starwest Botanicals, Sacramento, CA; HMF 6: *Boswellia* AKBA 5-Loxin, Pure Encapsulations, LLC., Sudbury, MA

§ Units of mg/g refer to products as fed

Of the five market formulations that had labels claiming an AKBA concentration, all of which also claimed to include BSE high in AKBA (HMF 1–3 and 6 and CMF 7), the mean AKBA concentration was 98.2 mg/g AF. For the five tested formulations with a label claim for AKBA, the mean percentage of detected AKBA was 173% of the label claim (range: 114–224%), with all of the market formulations containing more AKBA than the label claimed (Table 1).

Of the eight market formulations (HMF 4 & 5 and CMF 1–6) that did not have labels claiming an AKBA concentration, all except one (CMF 6) claimed to include BSE only (not BSE high in AKBA). The mean AKBA concentration of 7.4 mg/g AF in these supplements was significantly lower than the mean AKBA concentration of formulations claiming an AKBA concentration (98.2 mg/g AF) (*p =* 0.01).

## Discussion

### Validation of the derived method

The derived method used in the study was accurate for the analysis of AKBA and KBA from market formulations. This is supported by the lack of interfering peaks in the chromatograms, high accuracy as demonstrated by the mean recovery of 101.6%, high inter-assay and intra-assay precision, and linear calibration curves. In this study, a derivation of the previously validated method was used rather than that method itself in the hopes of using the same column and method to determine plasma concentrations of AKBA in dogs in a future clinical study. The mean recovery of 102.8±5.7% for AKBA and 100.3±3.1% for KBA is similar to a study by Shah that reported recoveries ranging from 94.1–105.9% [15]. Based on the results of this study, the authors propose that the validation methods utilized were acceptable.

### Analysis of the market formulations

The derived method accurately determined the concentrations of AKBA and KBA within the market formulations tested, and for formulations with AKBA concentration claims on the labels, all of the formulations contained greater concentrations than on the label with a range of 114–224% of the label claim. The higher detected values of AKBA within market formulations suggest that similar to guaranteed analyses on pet food labels, many of the concentrations of potentially active boswellic acids on nutraceutical labels may be more of a minimum value than an accurate concentration. It is therefore difficult to calculate an accurate dose based on the product label, as the dose administered may be significantly higher than that calculated from the label. It is unknown based on the findings of this study how similar the concentrations of KBA and AKBA are between batches of a specific nutraceutical product, and this should be evaluated in future studies.

A prior study determined the concentrations of AKBA and KBA within three supplements (2 tablets and 1 capsule) containing BSE to be: 3, 3.1 and 4.2% KBA (30, 31 and 42 mg/g AF) and 1, 1.5, and 2.6% AKBA (10, 15, and 26 mg/g AF) [15]. In comparison, of the eight market formulations (HMF 4 & 5 and CMF 1–6) in the current study with labels claiming BSE only (not high in AKBA), the concentrations of KBA and AKBA were generally lower at 0–2.47% (0–24.7 mg/g AF) and 0.01–2.35% (0.11–23.5 mg/g AF), respectively. Thus, this study confirms findings from prior studies that the concentrations of active boswellic acids within BSE are highly variable [15, 16]. In addition, to the authors’ knowledge, this is the first study to include market formulations produced for canine use, demonstrating that these also show a large variance. Even within the group of formulations claiming to contain “*B. serrata* gum resin extract”, though generally yielding higher concentrations of AKBA than other formulations, there remained a large variance in the concentration of AKBA (12.7–155.7 mg/kg AF). Therefore, attempting to provide a dose of active boswellic acids from nutraceutical products containing BSE as a source would be extremely difficult, if not impossible, without further testing of these products using validated methods such as described herein. In addition, when examining the market label claims of BSE it is evident that the dosing regimens suggested by manufacturers are well below the doses provided by Reichling and colleagues [10]. Currently, due to these manufacturers’ suggestions, underdosing of BSE is likely although conversely the potential for toxicity is likely low given that a concentrated AKBA product (Aflapin™) fed to rats had a no observed adverse effect level greater than 2500 mg/kg body weight [8]. Similar studies in dogs have not yet been performed. Therefore, the manufacturer labeled dosing suggestions appear anecdotal, and evidence-based dosing recommendations should be developed.

Currently, the authors are aware of only one non-placebo controlled study that has evaluated oral supplementation of BSE to dogs with naturally occurring osteoarthritis [10]. This study administered a BSE containing > 50% triterpenic acids at a dose of 40 mg of BSE per kg of body weight and demonstrated a reduction in clinical signs of naturally occurring osteoarthritis in supplemented dogs. While this study yielded favorable clinical results, the limitations of not including a placebo group, not providing dosages for the most biologically active triterpenic acids, and not demonstrating bioavailability, make it difficult to draw meaningful conclusions on the use of oral BSE supplementation in osteoarthritic dogs. However, these favorable results, combined with the number of BSE products currently on the market with purported benefits for osteoarthritic dogs, demonstrate the need for future clinical evaluation of the bioavailability, tolerability, dosing, and clinical efficacy of oral BSE supplementation in dogs.

## Conclusions

This study demonstrated a large variation of active boswellic acid concentrations in market formulations claiming to contain BSE, with products with a label claim for AKBA containing higher concentrations of AKBA than other products. The variation in boswellic acids across products confirms the need for third-party laboratories to quantify concentrations of active ingredients in market formulations containing BSE prior to the use of these formulations in the research or clinical setting, so that an appropriate clinical dosing regimen for conditions such as canine osteoarthritis can be developed. The amount of labeled boswellic acids on supplements labeled for humans and for pets should be regarded as a minimum concentration when calculating doses for dogs, and products with a label claim for AKBA should be used when calculating doses based on this compound.

## Methods

### Instrumentation & reagents

The HPLC system (PerkinElmer, Inc., Shelton, CT 06484, USA) consisted of a Series 200 autosampler, Series 200 LC Pump, and a Flexar UV/Vis detector. The column was a LiChrospher RP-18 (5 um, 125 mm × 4 mm, 100A) (Phenomenex, Torrance, CA 90501, USA) with a corresponding guard column (4 × 4 mm, replaced after 50–60 injections). Individual boswellic acid standards of primary analytical grade (Acetyl-11-Keto-β-Boswellic Acid, 3-(P) (AKBA) and Keto-β-Boswellic Acid, 11-(P) (KBA)) were purchased from ChromaDex (Irvine, CA 92618, USA). All chemicals and reagents used in the study were of high purity. HPLC grade methanol (MeOH), acetonitrile (ACN) and 85%-ortho-phosphoric acid were purchased from Fisher Scientific (Suwanee, GA 30024, USA). Ultra-pure water was produced using a Purelab Classic system (ELGA LabWater, High Wycombe HP14 3BY, UK).

### Chromatographic conditions

The mobile phase consisted of mobile phase A: H2O-MeOH-orthophosphoric acid (90:9.5:0.5, v/v) and mobile phase B: MeOH-ACN-H2O-orthophosphoric acid (55:40:4.5:0.5, v/v). The following gradient program was used: 0-10 min, 90% B and 10% A; 11–14 min, linear gradient change to 100% B; and 14-15 min, instant change to 90% B and 10% A. Samples were analyzed using the following parameters: 1.0 mL/min flow rate, 50 μL injection volume, and 250 nm detection wavelength.

### Calibration curve of standard AKBA and KBA

Accurately weighed AKBA and KBA standard powders were each mixed with MeOH and further diluted, yielding standard solutions for each acid ranging from 10 to 100 μg/mL. Spectrophotometry (Shimadzu, Columbia, MD 21046, USA) using 50 mg/mL dilution samples of each acid showed a maximum absorption for both acids at 250 nm. Fifty microliters from each standard solution was injected into the chromatographic system. The calibration curves for each acid were prepared by plotting peak areas of AKBA and KBA against respective concentrations. Combination AKBA and KBA standard solutions with a ratio of 1:1 and 9:1 were also prepared, injected into the chromatographic system and a calibration curve constructed, as described for the individual standard solutions. The 9:1 ratio combination standard solution was prepared in order to ensure the correct peaks were identified as AKBA and KBA in the combination standard chromatograms.

### Validation of the method

While this method was derived from a previously validated method, due to changes in instrumentation and chromatographic conditions, validation of this method was attempted in terms of linearity, accuracy, precision, and limit of detection. The linear responses for AKBA and KBA in the ranges of 10-100 μg/mL of each acid were assessed by correlation coefficient values.

The accuracy was determined by using three traditional Chinese veterinary herbal formulations (Wei Qi Booster, Stomach Happy, and Liver Happy), which have been previously analyzed [17]. These herbal formulations were additionally pre-analyzed to confirm the absence of interfering peaks. For each herbal formulation, 0.9 mg standard AKBA powder, 0.9 mg standard KBA powder, and 198.2 mg of a known herb were accurately measured and combined into a 30 mL beaker, yielding 200 mg sample powder with known concentrations of each boswellic acid. MeOH (12 ml) was added to the same beaker, and the mixture was sonicated for 30 min. Contents were filtered through Whatman Filter Paper (No. 1, 11 μm) into a 50 ml volumetric flask, with an additional 18 mL MeOH added to the flask, yielding a total of 30 mL MeOH (for a known concentration of 30 μg/ml of AKBA and KBA). The sample was then filtered through 0.45 μm syringe filters, injected into the chromatographic system, and quantities of AKBA and KBA were estimated, as described for the market formulations. This was repeated in triplicate for each of the three herbs listed above.

The inter-assay precision was determined by analyzing the combination standard solution in a ratio of 1:1 over the entire calibration range for 3 consecutive days. The intra-assay precision was determined by analyzing the combination standard solution in a ratio of 1:1 over the entire calibration range 3 times in the same day using the same calibration curve. For determinations of limit detection, the concentrations of standards lower than that of the lowest point of calibration curves were injected into the HPLC and responses were measured.

### Analysis of the market formulations

Thirteen market formulations were sampled, including six human and seven canine formulations (Table 1). All formulations, except CMF 6, were purchased through consumer retail channels. CMF 6 was available by prescription only and was therefore donated by the product manufacturer. The formulations were chosen based on searching common online search engines using the keywords “*Boswellia serrata* extract supplement” and “dog *Boswellia serrata* extract supplement”. The chosen formulations were deemed a representative sample by the authors considering the number of formulations available, formulation labeling and type of formulation (inclusive of tablets, chewable tablets, capsules, and powders). All of the human market formulations (HMF), except HMF 5, and CMF 7 were labeled to contain “*B. serrata* gum resin extract”. Of these, HMF 1 and CMF 7 were labeled to contain Aflapin™ and HMF 2 and 6 were labeled to contain 5-Loxin™. All of the canine market formulations, except CMF 3 and 7, were labeled to contain “*B. serrata* extract”. HMF 5 was a sample of pure frankincense (*B. serrata* resin) powder and CMF 3 was labeled to contain “*B. serrata*”.

Tablets were finely powdered and capsules were emptied. Tablet, capsule and powder equivalents of 100 mg BSE were accurately weighed, transferred to a 50 ml-volumetric flask and 20 mL MeOH was added. Each mixture was sonicated (Sonic Dismembrator 60, Fisher Scientific) for 30 min, diluted with an additional 30 mL MeOH then filtered through Whatman filter paper (No. 1, 11 μm), followed by ultrafiltration using 0.45 μm syringe filters. Human market formulations (HMF) 1–4 and 6 and all CMF were further diluted due to high concentrations of boswellic acids within these sample solutions.

The sample solution of each market formulation (50 μL) was injected into the chromatographic system three times and mean peak area of AKBA and KBA were recorded. The quantity of AKBA and KBA in each market formulation was estimated using the calibration curve created using the combined standard solutions with a ratio of 1:1. None of the market formulations had a label claim for KBA. When market formulation labels claimed an AKBA concentration, the amount of AKBA detected was divided by the label claim and provided as a percentage. The concentrations of AKBA detected in formulations claiming BSE only was compared to the concentrations of AKBA in formulations claiming BSE high in AKBA using a Mann Whitney test, with statistical significance assessed at a level of *p* < 0.05 (Minitab Express version 1.5.1).

## Data Availability

The datasets used and analyzed during the current study are available from the corresponding author on reasonable request.

## Abbreviations

ACN: Acetonitrile
AF: As fed
AKBA: 3-acetyl-11-keto-β-boswellic acid
BSE: *Boswellia serrata* extract
CMF: Canine market formulation
H2O: Water
HMF: Human market formulation
HPLC: High performance liquid chromatography
KBA: 11-keto-β-boswellic acid
MeOH: methanol
